# Shattering the Shield: Embracing Complexity in Undergraduate Medical Education

**DOI:** 10.1111/tct.70018

**Published:** 2025-01-20

**Authors:** Cara Bezzina, Robert McQuade, Wendy Lowe, Frances Mair, Lindsey Pope

**Affiliations:** ^1^ General Practice and Primary Care University of Glasgow Glasgow UK; ^2^ Centre for Medical Education, School of Medicine University of Dundee Dundee UK; ^3^ School of Medicine, Medical Sciences and Nutrition University of Aberdeen Aberdeen UK

**Keywords:** undergraduate medical education, complex patients, multimorbidity, reflexive thematic analysis, productive failure, experiential learning

## Abstract

**Background:**

Multimorbidity and patient complexity are increasing, yet undergraduate medical education curricula remain dominated by single disease frameworks, where students are often shielded from exposure to this complexity. Why this shielding continues to occur is understandable; however, this may leave graduates feeling underprepared for real‐world practice. This study aimed to explore medical students' experiences of encountering, managing and dealing with complexity and to provide informed recommendations for integrating complexity into clinical teaching.

**Methodology:**

Situated within a constructivist paradigm, this qualitative study involved focus groups (*n* = 4) with fourth‐ and fifth‐year medical students (*n* = 17) from two Scottish Universities. Data were analysed using reflexive thematic analysis.

**Findings:**

Learners in this study recognised multimorbidity, complex communication and emotionally charged interactions in their definitions of complexity. They described varying levels of exposure to complexity and opportunities to engage meaningfully with complex patients. Students felt that supervisors who shield students from learning opportunities with complex patients, together with a failing healthcare system, were critical limiting factors in their development. Learners emphasised the powerful role of supervisors in their learning experiences, which limited their ability to experiment and learn from productive failure but felt that with guided scaffolding and supervision, teaching and learning in this space could be meaningfully enhanced.

**Conclusion:**

Exposure to and engagement with complex patients offer critical learning opportunities that may allow students to explore and better develop skills in managing complexity. With appropriate scaffolding, students can be empowered to embrace complexity in the clinical learning environment, potentially equipping them to care for complex patients.

## 1 Introduction

Medical students are traditionally educated using single‐disease frameworks. However, in clinical practice, patients more often present with multiple issues. Multimorbidity, defined as the presence of two or more long‐term conditions, is increasingly becoming the norm [1]. Indeed, healthcare professionals are managing a growing number of patients with multiple chronic diseases alongside challenging psychosocial issues [2]. Consequently, although students are trained using single disease models, after graduation, they may find themselves thrust into clinical practice inadequately prepared for complexity that is not curated [3].

### 1.1 What Do We Mean by ‘Complex Patients’ and Why Explore Students' Experiences?

In this study, we explored the concept of ‘complex patients’ in medical education. A recent narrative review highlighted a lack of consensus in the literature on how best to define a complex patient [4]. Although complex patients are often regarded in healthcare as individuals with multiple diseases or health conditions [3], our approach goes beyond viewing the patient as a collection of conditions. Rather, we have adopted a ‘complex patient lens’ integrating both biomedical and psychosocial aspects of the patient's presentation [5].

Across the health service, we are witnessing an escalating level of complexity in clinical encounters [6]. With rising trends in multimorbidity [1], polypharmacy and consultation complexity [2], it is imperative our graduates develop the necessary generalist skills to care for these patients [7]. Addressing complex patients in undergraduate medical education is vital for several reasons. Firstly, given the prevalence of complex patients in clinical practice [6], it is inevitable that students and newly qualified doctors will encounter and learn from such patients during their placements and rotations. Secondly, the care of complex patients often requires collaboration from various healthcare professionals. Thirdly, we aspire to develop future graduates able to provide compassionate care that prioritises the individual needs, values and preferences of patients, also known as patient‐centred care [8]. This type of care requires an understanding of the patient's broader narrative, extending beyond a single disease focus.

### 1.2 What Do We Know About Complex Patients in Medical Education?

Clinical placements in medical school typically predominate later years of programmes, although variations exist depending on the country and specific curriculum. In the United Kingdom, medical students typically engage in experiential learning during their clinical placements in these senior years. This form of learning offers a dynamic and interactive approach, allowing students to explore and navigate complex situations while receiving guidance and feedback [9]. By engaging with real‐world experiences, learners connect theory to practice and deepen their understanding [10].

The ‘preparedness for practice’ literature focuses on the transition from medical school placements and experiential learning to clinical practice [11]. Studies investigating the preparedness of trainees for clinical practice identified a need for more comprehensive training in managing complex patients [12, 13]. Many newly qualified doctors feel overwhelmed when confronted with complex patient encounters [13], highlighting the importance of ensuring graduates are prepared for real‐life practice. However, when we delve into the literature exploring medical students' experiences with complex patients, the research predominantly focuses on students' experiences with specific patient groups, such as individuals currently in prison [14], non‐English speakers [15], veterans and refugee families [16].

This apparent disconnect between undergraduate medical education and the reality of clinical practice requires further exploration. This study aimed to enhance our understanding of how students learn from and engage with complex cases beyond single diseases or systems. Therefore, the central research question driving this study was, ‘What are medical students' experiences in relation to complex patients?’. We sought to gain insights into the students' readiness for clinical practice and identify specific challenges they faced in dealing with complex patients and complex patient encounters.

*This study aimed to enhance our understanding of how students learn from and engage with complex cases beyond single diseases or systems*.

## 2 Methods

This qualitative study used focus groups to explore the experiences of senior medical students about their learning in relation to complex patients. Throughout the study, we did not impose a definition on the students. Instead, we allowed them to interpret what constitutes a complex patient or patient encounter. To effectively investigate this phenomenon, a constructivist approach was chosen. Constructivism recognises that individuals create multiple realities, highlighting the importance of understanding subjective meanings and experiences of reality [17].

When considering medical educational theories that relate to complex patients, there are numerous options. Cognitive load theory, for example, considers the importance of simplifying materials based on students' capacities for processing information [18]. Alternatively, the social determinants of health, a conceptual framework that highlights the various factors that can influence individual and population health outcomes, could have been used as a lens to interpret this work [19]. Our study, however, takes a subjectivist inductive approach, gathering insights from focus groups rather than framing our work within a single theory or conceptual framework [20]. The study was undertaken in Scotland with undergraduate medical students attending two different medical schools (the University of Aberdeen and the University of Glasgow). Aberdeen provides a remote and rural program, whereas Glasgow students are typically based in urban clinical centres. The study therefore captures insights from students in varying learning environments.

### 2.1 Reflexivity

C.B. acknowledges her journey as a general practitioner (family physician) and educator influences her approach to understanding students' experiences. Her background, including experiences with complex patients and exposure to patient discrimination, shapes her perspective and drives this research. At the start of the interviews, participants were reassured that their views would remain confidential. Based on their openness in the focus groups, it seems evident that students felt comfortable sharing their vulnerabilities, experiences and opinions. C.B. also took steps to balance her viewpoint through supervisor discussions and joint coding, while also valuing the insights gained from her unique position and experiences with students.

### 2.2 Ethical Approval

The research design and protocol received approval from the Dundee ethics committee (SMED REC Number 22/138) and the University of Aberdeen ethics board (SERB/2021/12/2238). Gatekeeper approval was also secured from the University of Glasgow.

### 2.3 Data Collection and Analysis

Four focus groups were conducted by C.B. A total of 17 senior medical students participated: 11 in Aberdeen (nine women, two men) and six in Glasgow (three women, three men). All fourth‐ and fifth‐year Aberdeen students on placement in Inverness at the time of recruitment were invited to participate. In Glasgow, all fourth‐ and fifth‐year students were invited through an email distributed by the administrators to the entire cohort. The focus groups were audio recorded, with participant consent, and transcripts analysed using reflexive thematic analysis (RTA) by C.B. with a subset of the dataset also double coded by W.L. and R.M. RTA embraces reflexivity, subjectivity and interpretation and recognises these elements as an asset in the production of knowledge [21]. During the data familiarisation stage, C.B. transcribed the initial two focus group interviews and the latter two focus groups were professionally transcribed. To facilitate familiarisation, visual maps of each recording were created. C.B. implemented a meticulous journaling process to enhance reflexivity [21]. This process of self‐reflection allowed C.B. to foster critical reflection and embrace personal biases.

Postinterview debriefs with W.L., L.P. and R.M. played a pivotal role in stimulating critical self‐reflection regarding the researcher's active role in coconstructing knowledge. Regular meetings were held to discuss progress and incorporate feedback, ensuring transparency and enriching the analysis and reporting of the findings. The transcripts were coded manually in Microsoft Word, where comments served as notes, and journal entries were examined for additional context. This coding approach allowed for the extraction of coded excerpts to be used in theme construction. Given the exploratory nature of the research on students' experiences, an inductive approach was selected, as there was no pre‐existing theoretical foundation [21, 22]. The resulting codes were compiled for each interview, offering a comprehensive overview of the data. The researcher followed the approach described by Braun and colleagues [21] to develop the initial candidate themes, utilising codes as building blocks. Initially, the focus was on semantic codes like ‘definition of complexity’. As analysis progressed, deeper underlying meanings were generated, leading to a second coding round that refined the codes based on analytic notes and our research question, resulting in approximately 350 codes for further analysis. Theme development was facilitated through physical clustering of codes and iterative rearrangement. Notable patterns and similarities between the codes were observed. Codes provided supporting evidence for themes, whereas sub‐themes were identified to capture distinct aspects within a central organising concept. Themes that were redundant or overlapped were either removed or integrated into other parts of the analysis.

## 3 Findings

**FIGURE 1:**
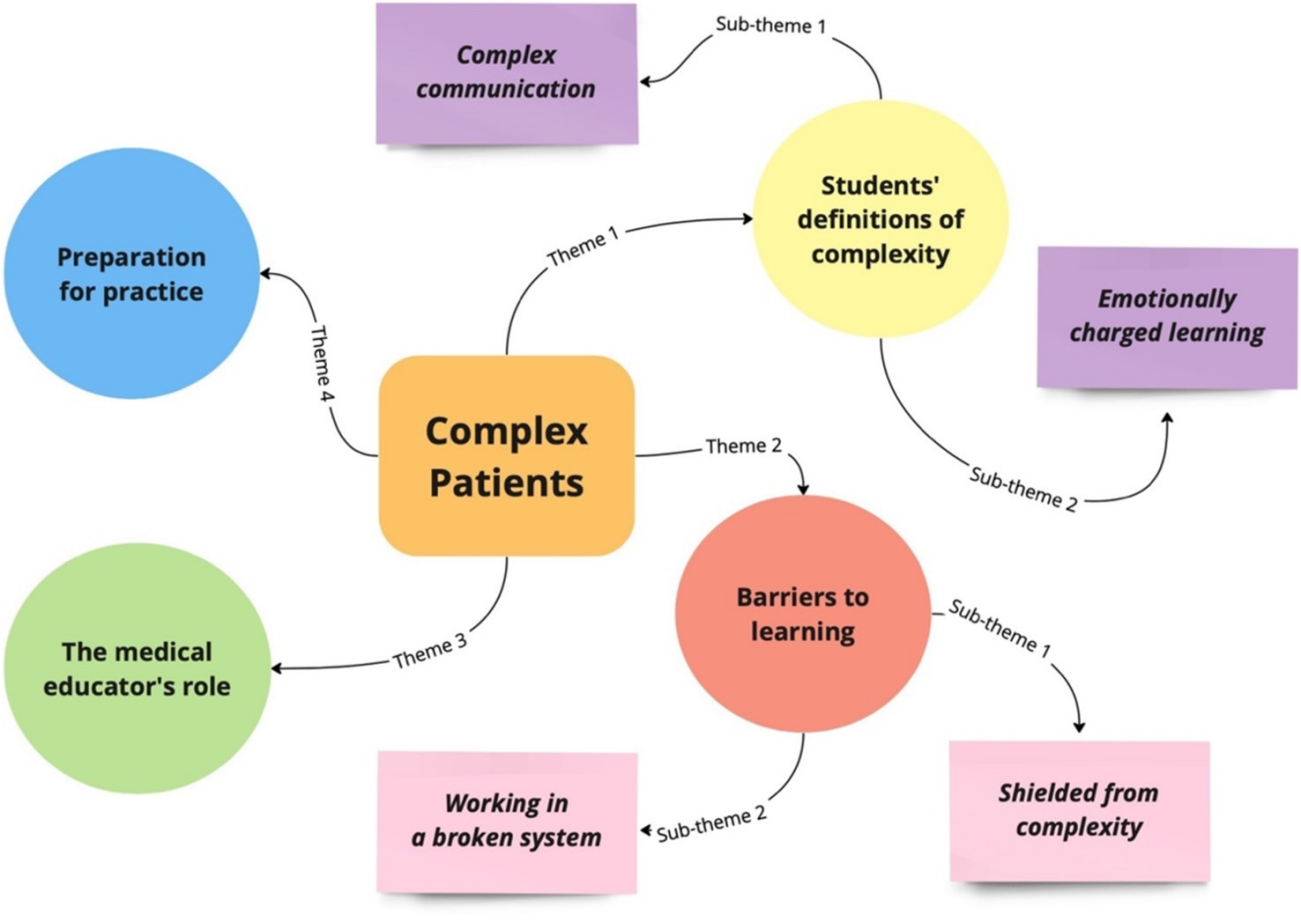
Outline of generated themes.

Four main themes were identified: (1) student definitions of complexity; (2) barriers to learning; (3) the medical educator's role; and (4) preparation for practice, of which two had sub‐themes. Figure 1 provides a mind map outlining these results.

### 3.1 Students' Definitions of Complexity

Participants shared a wide range of definitions for ‘a complex patient’, but there were consistent similarities across focus groups. For example, the biopsychosocial model was evident in all interviews, with several students mentioning this model within the context of multimorbidity. Some students challenged the entire notion of complexity, debating that there is no such thing as a simple or a complex patient. Others described the increasing complexity of cases encountered as they progress through training, simultaneously developing their ability to deal with complexity and comfort dealing with uncertainty. For some students, difficulty dealing with uncertainty defined complexity:

… You do not know what you are dealing with or who you are dealing with. Questions come up as you go in […] There's lots of complexity that comes up and takes me by surprise. That's tough.

A4M5

Despite the vast spectrum of definitions offered, there were two collections of interpretations that were most prominent: *complex communication* and *emotionally charged learning*.

#### 3.1.1 Sub‐Theme 1 *Complex Communication*

Students identified encounters with patients who had additional communication needs—such as language barriers and sensory impairments—as complex. These communication barriers were seen to create frustration and disconnection in the consultation. Students felt this complexity could result in prejudice towards some patients:

… I've seen it on wards where there have been deaf or hard of hearing patients, ‘Oh we'll ignore them, they are not going to hear what we have to say anyway’. And staff make decisions for them without asking them or spending time with them ….

G5F1

#### 3.1.2 Sub‐Theme 2 *Emotionally Charged Learning*

This sub‐theme focuses on the emotional reactions of students when discussing complexity, with most examples associated with negative emotions. Students used words such as ‘disillusioned’ and ‘demoralising’ to express how they feel about caring for complex patients in their future NHS careers. All students described difficulty unpacking and dealing with emotionally charged encounters. In this example, a student describes her experience with a mother who did not speak English:

… I've never been in a situation like that, but I cannot imagine how terrified she must have been. And all I could do to help her was give her my hand. I think that that did help her to some extent but the fact that that's all you can do, and you have just got to kind of watch someone suffer ….

G5F2

### 3.2 Barriers to Learning From Complex Patients

Many participants identified barriers preventing them from learning effectively from interactions with complex patients. These include the gap between medical school teaching and the real world, working in a system that does not cater to complex patients' needs and fighting against time. Participants expressed a clear desire and need for better teaching that reflects the reality of clinical practice and addresses the nuances of patient care. They described feeling flustered when faced with complex patients on placement, largely because they felt medical school taught them to approach patients through a narrow disease focus rather than the entirety of a patient's condition. This is reflected in the following quote:

I think it feels a bit overwhelming because in medical school you are told like a patient comes in with these classical symptoms. Then you go to clerk someone in and you start thinking about one aspect of history, but then you have other bits, and it just adds more and more and more to it. You get to a point where there's just so much there, and I'm used to dealing with everything with bite sized chunks, rather than combining a lot of things together. We have not had a huge amount of practice on that […] because when we do learn, we learn everything in isolation, but real patients aren't like that.

A4F2

From all the barriers shared by students, two key sub‐themes reflect the most significant challenges students face in their training: that they feel *shielded from complexity* and believe they will be *working in a broken system*.

#### 3.2.1 Sub‐Theme 3 *Shielded From Complexity*

Participants explained that they often feel shielded from complex cases and are only exposed to carefully selected examples of complexity, limiting their ability to develop the skills to handle challenging situations. A student reflected on this in a hospital setting:

I do not want to talk for everyone, but I think my involvement in it is quite limited. If you are on a ward round, you see a lot of patients that are very complex but if you were to ask the doctors, ‘Who's a good patient to go talk to? To take a history from?’, they tend to point you away from those people and go to more simple cases that are very cut and dry ….

G4M1

They felt that the system and/or educators tend to ‘protect’ them from dealing with complex patients. More than one student perceived that dealing with complexity is reserved for consultants or general practitioners, with students only allowed to observe from afar. Their feeling on the matter is exemplified in the quote below:

… But then you just kind of felt a bit powerless against it because you yourself could not do anything to make that situation better ….

A5F1

#### 3.2.2 Sub‐Theme 4 *Working in a Broken System*

Students feel burdened by the weight of the failing system, despite it not being their fault. They observed doctors struggling to provide holistic care due to the system's perceived inability to address complexity, resulting in patients' needs not being met. When discussing complex encounters, students often referred to cases that ‘keep a doctor up at night’:

I think it is demoralising. Like that case follows you home because you will be worrying about them and, you know it's not your fault. As the doctor you are the representation of the health care system, and that health care system is obviously failing ….

A4M5

### 3.3 The Medical Educator's Role

This major theme focuses on the impact of medical educators on the student's learning experience. Participants often perceived they had contrasting responses to complexity to their supervising clinicians, highlighting the significance of role modelling in response to challenging or complex situations:

… And I just kind of walked out, took a deep breath and I was like ‘Oh my gosh, this is not the type of doctor I want to be’.

G5M1

Students often do not disclose these difficult encounters with supervisors, citing the lack of a safe space for discussing their experiences. This appeared to be further compounded by the fact that they may lack a consistent point of contact or supervisor who is willing to debrief. Learners stated that supervisors vary greatly in their willingness and ability to support them in processing these experiences as exemplified below:

… It's quite daunting … A lot of the time, I've never even met my block lead. I've not had a consistent person who would talk to me. I just show up every day and try and tag along with someone. So, I think it's hard if you are trying to talk about how, you feel as it's a very vulnerable situation ….

A4F7

### 3.4 Preparation for Practice

Although some students believed they were ready for practice, others expressed concerns about their education and training. One student acknowledged that although they felt prepared, their training did not appear to intentionally include complex patient management. Students expressed that they find it challenging to learn from doctors who may not be skilled at teaching about complexity.

Just because someone's a qualified doctor does not necessarily mean they are qualified to teach you about complexity.

G4M1

Other participants agreed that dealing with complex patients was inherently difficult and challenging, but they still felt they would cope by simply taking a clinical history in the usual way. Many students hoped that, with experience, their ability to manage complex patients would improve:

I do not think I'll ever feel ready … because complexity is, well, complex. [Laughter]. I feel like with experience it will get better … There will be complex patients which I'm not sure what's going on but I will feel more ready, and hopefully we will have the support to be able to deal with it.

A5F4

## 4 Discussion

The aim of this study was to explore how senior medical students engage with and learn from complex patients. Key findings reveal substantial challenges faced by students in clinical education, notably because of a strained healthcare system and supervisors often shielding students from complex patient cases, thereby limiting their experiential learning. Students defined complexity as involving challenging communication, emotional patient interactions, and the presence of multiple health issues. They expressed a strong interest in receiving more training for these demanding situations yet expressed mixed feelings about their readiness for real‐world clinical work. The central message that became clear from our discussions with students is that we must better prepare them to embrace complexity earlier within the CTE.

Our study participants were acutely aware of the challenges confronting the National Health Service in the United Kingdom [23]. This may resonate with students in other health systems. The articulation of these concerns during educational interviews underscores the gravity and impact of this issue. It is paramount to acknowledge the realities of the struggling health system within which patients, health professionals, educators, and students are deeply embedded. Furthermore, it is important to recognise that as clinicians become busier in their practice, being an effective teacher becomes more challenging because of limited time for education [24].

Despite these challenges, clinical placements offer students experiential learning opportunities that are essential for their development as doctors [9]. In our study, we heard learners describe their experiences with complex patients as potentially overwhelming and challenging. This could be compounded by a perceived lack of support in these situations. The predominantly negative experiences reported by students also raise questions about the hidden curriculum experienced by students and the learning culture within their learning environments, especially in relation to learning from and caring for complex patients [25]. Students recounted instances of witnessing negativity and dismissive behaviour from senior healthcare professionals towards complex patients who had a disability or did not speak English, promoting concerns about potential biases [26]. Although the personal opinions and experiences of clinicians undoubtedly play a role, it is essential to consider other potential influencing factors. Students pointed out that physicians may experience fear or anxiety when dealing with complex patients or may attempt to distance themselves from challenges they perceive as insurmountable. Indeed, supervisors may experience their own uncertainty when encountering complex patients however how they navigate this will be interpreted by learners in different ways, including role modelling positive or negative behaviours, as evident in our study.

### 4.1 Navigating Complexity With the Right Support

Yardley et al. argue that, without proper support, learners may underestimate the educational value of experiences or be confused by dissonances between experiences in different contexts [27]. It was surprising that students did not share more experiences around dealing with multimorbidity or polypharmacy. It could be hypothesised that this is due to the Dunning–Kruger effect [28], where students do not know what they do not know. This is where educators' perspectives could have been invaluable. Indeed, the findings of this study provoke questions about whether students should be shielded from complex patients or actively encouraged to engage with them under the guidance of a facilitator. Currently, it seems that learners are either being ‘shielded’ or ‘thrown into the deep end’, with limited support in between [29].

Although clinicians may feel compelled to ‘shield’ students from complex clinical encounters, research shows that experiences that evoke discomfort or present unexpected challenges can prompt students to question established practices, foster the development of their professional identity and prepare them for independent practice in a continuously evolving profession [30, 31]. When learners are confronted with complex problems, it is not uncommon for them to experience a sense of bewilderment [32]. In such situations, educators often have the natural inclination to swiftly come to their rescue, aiming to prevent them from feeling lost or inadequate. However, psychologists have observed a pattern among individuals who possess an open mindset—they tend to respond to confusion with curiosity and interest [33]. Students need to be given the support, space and time to foster this curiosity. Rather than shying away from encouraging students, educators should strive to be learner‐centric and embrace and acknowledge the cognitive dissonance and perplexity that arise during these learning encounters. It is crucial to consider whether students' perceptions of supervisor‐imposed limitations arise from their own insecurities or are reflective of the broader expectations of supervisors. There is a risk of oversimplifying this multifaceted issue to a mere assignment of blame on supervisors, which is not our intention. Nonetheless, this remains a learner‐centric study, and we present an exploration centred on the student's perspective.

### 4.2 Preparing for Real‐World Challenges

**FIGURE 2:**
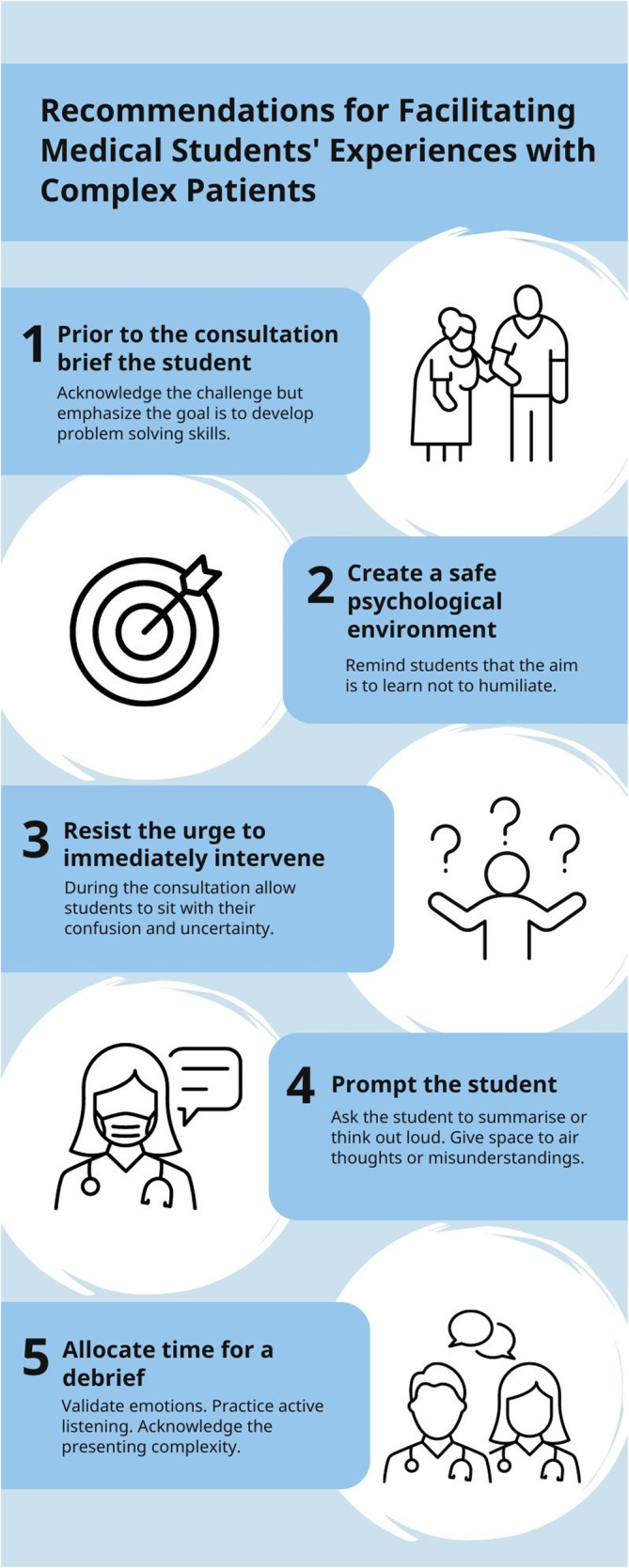
Summarisation of recommendations for facilitating medical students' experiences with complex patients.

Acknowledging the complexity of clinical practice is crucial for adequately preparing medical students for their future roles. It is clear from this study that students are aware of the disparity between classroom learning and the realities of clinical work. Simplifying or streamlining complexity to meet predefined learning outcomes can leave learners ill‐prepared to navigate real‐life patient scenarios [12]. Therefore, our proposed response to complexity is not a complex solution but rather a simple one. Rather than seeking radical new ideas, this work demonstrates a reorientation towards fundamental principles, such as the doctor–patient relationship. This study highlights the importance of prioritising this interaction, urging supervisors to allocate time for discussing complex patient encounters with their students. By bringing this fundamental teaching opportunity to the forefront and facilitating guided reflection on these experiences, educators are more likely to equip students with the necessary skills to handle complexity effectively. Figure 2 is based on our findings and aims to assist clinical educators guide students through complex clinical encounters, summarising our recommendations.

*Acknowledging the complexity of clinical practice is crucial for adequately preparing medical students for their future roles.*

Creating a safe learning environment that encourages students to identify and discuss their misunderstandings is paramount [34]. By gradually exposing students to complex clinical scenarios under the guidance of their supervisors, educators can better equip them for the challenges they will face as doctors. Students were clear that the presence of a senior clinician during these situations provided them with a sense of safety and encouraged them to engage with patients. Therefore, a key recommendation offered is for tutors and supervisors to cultivate a supportive and inclusive learning environment where students can be challenged to engage in productive failure [35]. This educational approach encourages learners to solve a problem without guidance, allowing them to grapple with the challenge with minimal initial guidance, resulting in deep engagement, better understanding and greater retention of learning [32].

*Creating a safe learning environment that encourages students to identify and discuss their misunderstandings is paramount*.

### 4.3 Strengths and Limitations

We encouraged learners to describe what they experienced as a complex patient or encounter as individuals may find different cases complex based on their prior skills and experience, and this provided a broad representation of complexity. Despite the differences in curriculum and the contextual factors associated with each university, the results of the study demonstrated commonality in the experiences reported by participants, suggesting that the identified themes and challenges are likely to be prevalent across different settings. Although qualitative research is not intended for statistical sample generalisability, one can certainly identify trends and patterns in a rich dataset [21]. However, the findings of this study are situated within its specific context, in a single country (Scotland), and thus should be interpreted accordingly. In addition, the recruitment of participants in Glasgow posed challenges, with limited engagement from a larger class. It is worth noting that the self‐selection process employed for recruiting students for the focus groups may have attracted participants who were eager to share their experiences, potentially resulting in a sample that is already well‐informed about the challenges associated with caring for complex patients. Finally, this study does not include insights from educators, junior doctors or patients, which could have provided valuable additional perspectives. This limitation stems from the study being part of a Master's program with constraints on time and resources.

## 5 Conclusion

In conclusion, this study has identified critical issues that influence students' experiences in the clinical teaching environment. Learners desired enhanced teaching and learning approaches tailored explicitly to interacting with complex patients. The findings illuminate numerous challenges faced by learners but also highlight the challenges faced by complex patients within the healthcare system. This work also spotlights the significant role of supervisors in shaping student experiences, often shielding them from engaging with complex patients and limiting their exploration and learning from productive failure. Our work emphasises the importance of fostering an educational culture that encourages active engagement with complex patients, calling for a cultural transformation within the clinical teaching environment. Further research is required to better understand educator's perspectives and develop appropriate frameworks to promote better education in relation to complex patients. As medical educators, we have the opportunity and the imperative to stand at the forefront of this paradigm shift. So, the real question is—why do educators shield students from complexity?

*Learners desired enhanced teaching and learning approaches tailored explicitly to interacting with complex patients*.

## Author Contributions

**Cara Bezzina:** conceptualization, investigation, funding acquisition, writing – original draft, methodology, validation, writing – review and editing, formal analysis, project administration, data curation, resources, visualization, software. **Robert McQuade:** conceptualization, investigation, methodology, validation, writing – review and editing, supervision, formal analysis, project administration, writing – original draft. **Wendy Lowe:** conceptualization, investigation, writing – review and editing, formal analysis, supervision, methodology. **Frances Mair:** funding acquisition, writing – review and editing, project administration, supervision, writing – original draft. **Lindsey Pope:** conceptualization, investigation, methodology, funding acquisition, writing – original draft, visualization, writing – review and editing, validation, formal analysis, supervision, resources.

## Ethical Approval

The research design and protocol received approval from the Dundee ethics committee (SMED REC Number 22/138) and the University of Aberdeen ethics board (SERB/2021/12/2238). Gatekeeper approval was also secured from the University of Glasgow.

## Consent

We confirm that all participants included in this study provided informed consent before participating. Participants were informed about the purpose, procedures, risks and benefits of the study. Participation was voluntary, and participants were assured of confidentiality.

## Conflicts of Interest

The authors declare no conflicts of interest.

## Data Availability

The data supporting this study are not publicly available because of University Regulations and confidentiality procedures. Anonymized transcripts are accessible upon request, subject to ethical approval.
